# Effect of Obesity on Surgical Outcomes and Complication Rates in Pediatric Patients: A Comprehensive Systematic Review and Meta-Analysis

**DOI:** 10.7759/cureus.54470

**Published:** 2024-02-19

**Authors:** Yeisson Rivero-Moreno, Andrea Garcia, Miguel Rivas-Perez, Jesus Coa-Bracho, Yoalkris Salcedo, Gabriel Gonzalez-Quinde, Erinor Montero-Palma, Denisse Valdivia-Sepulveda, Marialejandra Paz-Castillo, Debbye Machado-Paled, Wilson Garcia-Cazorla, Katheryn Acero-Alvarracín, Laila Tarabey-Yunis, Cesar Estrella-Gaibor

**Affiliations:** 1 Department of Surgery, Universidad de Oriente, Anzoategui, VEN; 2 Department of Internal Medicine, Universidad de Oriente, Ciudad Bolivar, VEN; 3 Department of Surgery, Hospital de Talagante, Santiago de Chile, CHL; 4 Department of Surgery, Universidad Iberoamericana, Santo Domingo, DOM; 5 Department of Surgery, Hospital Leon Becerra Camacho, Milagro, ECU; 6 Department of Surgery, Centro de Estudios Universitarios Xochicalco, Xochicalco, MEX; 7 Department of Surgery, Universidad Catolica de Honduras, Tegucigalpa, HND; 8 Department of Surgery, Universidad de Cuenca, Cuenca, ECU; 9 Department of General Surgery, Universidad de Guayaquil Facultad de Ciencias Médicas, Guayaquil, ECU; 10 Department of Surgery, Universidad Centroccidental Lisandro Alvarado, Lara, VEN; 11 Department of General Surgery, Ministerio de Salud Pública, Hospital Esmeraldas sur Delfina Torres de Concha, Quito, ECU

**Keywords:** operative time, length of stay, postoperative abscess, deep venous thromboembolism, wound dehiscence, surgical site infection, surgical complication, risk factor, pediatric obesity

## Abstract

Obesity is one of the primary public health problems faced by children. The increased incidence of obesity in the pediatric population poses significant challenges during and after surgical procedures. This systematic review and meta-analysis aimed to understand to what extent obesity is to surgical complications in pediatric patients. A systematic database search of PubMed, Web of Science, Scopus, and Science Direct was performed in June 2023. According to the inclusion and exclusion criteria, two evaluators independently conducted literature screening, data extraction, and quality evaluation of the retrieved literature. The Newcastle-Ottawa Scale score was used for quality evaluation, and a meta-analysis was performed using Review Manager software 5.4.1. A total of 1,170 relevant articles were initially identified, and 20 articles were finally included for data extraction and meta-analysis. The results of the meta-analysis showed that compared with normal-weight individuals, obese pediatric patients had a higher risk of developing surgical site infection (SSI) (relative risk (RR) = 1.63; 95% confidence interval (CI) = 1.33-2.00), wound dehiscence (RR = 2.01; 95% CI = 1.24-3.23), and underwent procedures that were 11.32 minutes longer (95% CI = 5.36-17.29). There were no differences in bleeding requiring transfusion, deep venous thromboembolism, postoperative abscess rate, and length of stay. Obese pediatric patients have a higher risk of SSI and dehiscence, along with a longer operative time. The established risks in obese adults undergoing surgery suggest a similar risk for obese pediatric patients. The findings of this study hold significant implications for clinical practice, suggesting the potential for additional measures to prevent surgical complications in children.

## Introduction and background

Obesity and overweight is a complex, multifactorial, and largely preventable disease affecting over a third of the world’s population today. According to the 2023 World Health Statistics Report from the World Health Organization (WHO), the age-standardized prevalence of obesity among adults aged 18 years or older has been rising since the 1970s. In 2016, 13.1% of adults globally were obese, up from 8.7% in 2000 [1]. If these trends continue, by 2030, an estimated 38% of the world’s adult population will be overweight, and another 20% will be obese [2].

Childhood obesity is one of the primary public health problems faced by children. Data from WHO reported that globally in 2000, 33.0 million children under five years of age (5.3%) were overweight while in 2022 this number had risen to 37.0 million [1]. According to the National Health and Nutrition Examination Survey, in the United States, from 1999 to 2016, 18.4% of children ages 2-19 years were obese [3]. Furthermore, in the United Kingdom, it has been reported that around one-third of children aged 2-15 years are overweight or obese and that this proportion is likely to increase in the future [4].

It is well acknowledged that overweight and obesity are risk factors in adult patients, associated with numerous potential comorbidities, including respiratory disorders, high blood pressure, and diabetes [5]. Surgical procedures in these patients tend to be more complex owing to their greater technical and anesthetic difficulty as anatomical references are more complicated to visualize, difficult airways are more frequent, and aspiration risk is higher due to a greater gastric residual volume. The increased incidence of obesity in the pediatric population poses significant challenges during and after any surgical intervention. However, the literature on the effects of overweight and obesity in pediatric abdominal surgery is scarce compared to that of adult patients [6].

Several studies have reported obesity as a risk factor for poor surgical outcomes after different types of interventions [7-9]. Still, they evaluated the impact of obesity in a specific type of surgery. Some meta-analyses have gathered data but remain focused on certain complications for a specific surgical procedure [10].

No study has evaluated or synthesized the possible extent of this risk for different complications or after a surgical procedure in general, which makes it essential to understand how obesity in the pediatric population could impact any surgical intervention. This systematic review and meta-analysis aimed to understand to what extent obesity could be related to surgical complications in pediatric patients, providing valuable insights into the risk factors for those complications, as well as improving patient outcomes. Furthermore, this study can inform future research in the field and guide the development of evidence-based guidelines for preventing such complications.

## Review

Methodology

Study Design

A systematic review and meta-analysis was conducted to investigate how obesity could be related to surgical complications in pediatric patients. We registered the protocol of this systematic review in the International Prospective Register of Systematic Reviews (PROSPERO) (CRD42023424447). We conducted it according to the Preferred Reporting Items for Systematic Review and Meta-Analysis Protocols (PRISMA-P 2020) guidelines [11]. We did not seek formal ethical approval as we used data from published primary studies.

Search Strategy, Data Sources, and Screening

A comprehensive search strategy was developed to search the following databases: PubMed, Scopus, Web of Science, and Science Direct. The search was last performed on May 19, 2023. The search terms included derived from the keywords “pediatric obesity” and “surgical complications.” Medical Subject Heading (MeSH) terms were used along with synonyms. A complete list of search terms can be found in Table 1.

**Table 1:** Search terms for the meta-analysis in PubMed, Scopus, Web of Science, and Science Direct databases.

Database	Search terms
PubMed	((“Child”[MeSH Terms] OR “child, preschool”[MeSH Terms] OR “Adolescent”[MeSH Terms]) NOT “Adult”[MeSH Terms]) AND (“Obesity”[MeSH Terms] OR “Pediatric Obesity”[MeSH Terms] OR “obesity, abdominal”[MeSH Terms]) OR “Pediatric Obesity”[Title/Abstract] OR “Obesity in Childhood”[Title/Abstract] OR “Childhood Onset Obesity”[Title/Abstract] OR “Child Obesity”[Title/Abstract] OR “Childhood Obesity”[Title/Abstract] OR “Adolescent Obesity”[Title/Abstract] OR “Obesity in Adolescence”[Title/Abstract] OR “Infantile Obesity”[Title/Abstract] OR “Infant Obesity”[Title/Abstract] OR “Childhood Overweight”[Title/Abstract] OR “Infant Overweight”[Title/Abstract] OR “Adolescent Overweight”[Title/Abstract] AND (“Intraoperative Complications”[Mesh] OR “Postoperative Complications”[Mesh] OR “Peroperative Complication”[Title/Abstract] OR “Peroperative Complications”[Title/Abstract] OR “Intraoperative Complications”[Title/Abstract] OR “Intraoperative Complication”[Title/Abstract] OR “Surgical Injury”[Title/Abstract] OR “Surgical Injuries”[Title/Abstract] OR “Postoperative Complication”[Title/Abstract] OR “Postoperative Complications”[Title/Abstract])
Scopus	(“Child” OR “child, preschool” OR “Adolescent” AND NOT “Adult”) AND (“Obesity” OR “Pediatric Obesity” OR “obesity, abdominal” OR “Obesity in Childhood” OR “Childhood Onset Obesity” OR “Child Obesity” OR “Childhood Obesity” OR “Adolescent Obesity” OR “Obesity in Adolescence” OR “Infantile Obesity” OR “Infant Obesity” OR “Childhood Overweight” OR “Infant Overweight” OR “Adolescent Overweight”) AND (“Intraoperative Complication” OR “Postoperative Complication” OR “Peroperative Complication” OR “Surgical Injury”)
Web of Science	TS=(Child OR child, preschool OR Adolescent NOT Adult) AND TS=(Obesity OR Pediatric Obesity OR obesity, abdominal OR Obesity in Childhood OR Childhood Onset Obesity OR Child Obesity OR Childhood Obesity OR Adolescent Obesity OR Obesity in Adolescence OR Infantile Obesity OR Infant Obesity OR Childhood Overweight OR Infant Overweight OR Adolescent Overweight) AND TS=(Intraoperative Complication OR Postoperative Complication OR Peroperative Complication OR Surgical Injury)
Science Direct	(Child OR child, preschool OR Adolescent NOT Adult) AND (Obesity OR Pediatric Obesity OR Childhood Overweight OR Infant Overweight OR Adolescent Overweight) AND (Intraoperative Complication OR Postoperative Complication OR Peroperative Complication OR Surgical Injury)

Two groups of independent reviewers screened the titles and abstracts of identified studies for relevance based on the inclusion and exclusion criteria. The full text of potentially relevant studies was then assessed for eligibility. Discrepancies between reviewers were resolved through discussion or consultation with a third reviewer.

We included cohort, case-control, and cross-sectional studies that compared the incidence of surgical complications in pediatric patients according to their weight (normal vs obese). We excluded studies in which the surgical procedures were related to weight loss surgery and those with overlapping populations, such as studies from different periods from the same database. There were no restrictions on the date of publication and language.

Our study population was pediatric patients between 2 and 18 years of age who had undergone any surgical procedures considering pediatric obesity as the exposition. According to WHO, pediatric obesity is a weight-for-height greater than two or three standard deviations above the child growth standards median in children under 5 years or between 5 and 19 years, respectively [12]. The United States Centers for Disease Control and Prevention of the United defines obesity as a body mass index (BMI) in the 95th percentile or greater [13]. The comparison group was pediatric patients with normal weight. Patients who were underweight or overweight were not considered. The primary outcomes evaluated were surgical wound dehiscence, surgical site infection (SSI), including superficial and deep wounds, bleeding that required transfusion, deep venous thromboembolism (DVT), and postoperative abscess formation as defined by individual studies [14,15]. These outcomes were selected as they are the most commonly studied in different meta-analyses that evaluate the effect of obesity on surgical outcomes within the adult population. Time of surgery and length of stay (LOS) at the hospital were also evaluated.

Data Extraction

We extracted the following characteristics from the studies: authors, year of publication, country, study design, sample size, follow-up period, and type of surgical intervention. Patient characteristics included age, sex, number of normal-weight and obese patients, and the frequency of the evaluated outcomes.

Quality Assessment

Two reviewers used the Newcastle-Ottawa Scale (NOS) specific for cohort studies to assess the quality of the included studies. The scale scores range from 0 to 9, where 0 is the poorest quality and 9 is the highest [16].

Statistical Analysis

A systematic review with a subsequent meta-analysis was conducted using Review Manager software 5.4.1 (The Cochrane Collaboration, 2020) to estimate the overall effect size of obesity on the incidence of surgical complications in pediatric patients. Statistical heterogeneity was analyzed with I^2^ and Tau^2^, and the funnel plot analysis was performed to assess publication bias. Proportions were used to present frequencies, mean difference was used to compare continuous data using the inverse variance statistical method, and the relative risk (RR) was used to compare dichotomous outcomes under the Mantel-Haenszel statistical method, both with 95% confidence intervals (CIs) and applying a random-effects model to report the results. Additionally, a subgroup analysis was performed based on the field of surgery (general surgery, neurosurgery, and orthopedic surgery, among others). Subgroup analyses were included when there were two or more studies to compare for each surgical subspecialty.

Risk of Bias Across Studies

We assessed heterogeneity for each outcome using I^2^ and defined I^2^ of 75% and greater as substantial heterogeneity, 25%-75% as moderate heterogeneity, and below 25% as low heterogeneity, as defined by Higgins et al. [17]. We used a comparison-adjusted funnel plot and Egger’s test to evaluate the risk of publication bias. An asymmetrical funnel plot and a p-value <0.1 on Egger’s test indicated the presence of publication bias [18].

Results

Study Selection

**Figure 1:**
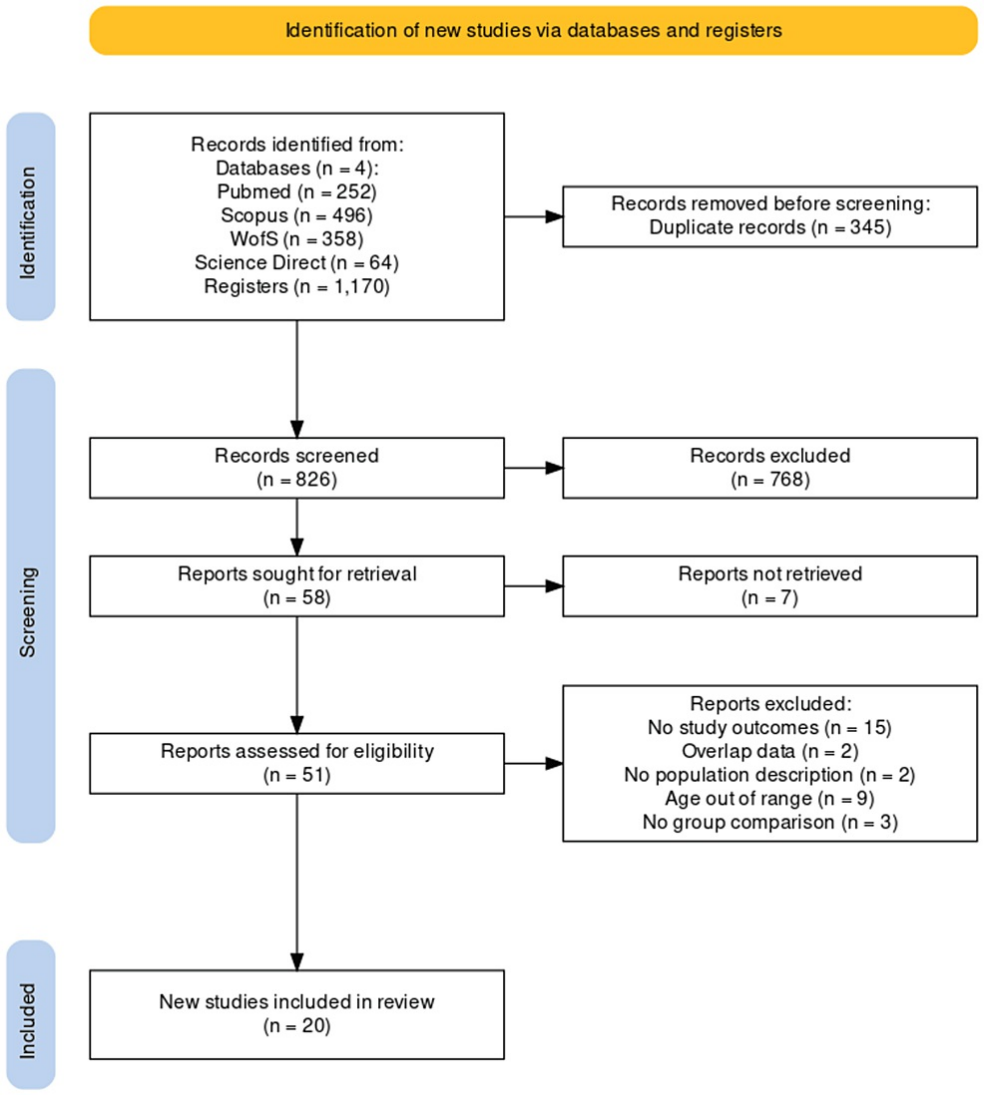
Search outputs based on Preferred Reporting Items for Systematic Review and Meta-Analysis guidelines.

From four different databases, we identified 1,170 records in the initial literature search, of which 345 were duplicates. We conducted title and abstract screening of 826 records and selected 58 studies for full-text review based on predetermined inclusion and exclusion criteria. Seven studies were excluded because they could not be retrieved. After a full-text review, we finally extracted data from 20 articles, all published in English. Reasons for final exclusion and the flowchart of the study selection are detailed in the PRISMA flowchart of this study presented in Figure 1, which was structured through the Haddaway et al. online app [19].

Study Characteristics

The 20 included studies were published between 2005 and 2023, with a median of 2017 (CI = 2014-2019). All of them were cohort studies that gathered the data from four years as a median (CI = 2-6). Among studies that specified the follow-up period, nine (40.9%) were 30 days, and three (13.6%) were one to five years. The remaining 11 did not indicate the follow-up period. Most studies were from the United States (70%), followed by Canada (10%), and the rest from China, Italy, Spain, and Taiwan (5% each). To assessm the effect of obesity on surgical outcomes, studies based on different surgical procedures were included. The frequency of those in descending order was general surgery with 12 (34.1%) studies, neurosurgery with six (17%), orthopedic surgery with seven (20%), urology with three (9%), cardiothoracic surgery with two (6%), otorhinolaryngology surgery three (9%), and plastic surgery with two (6%). Concerning general surgery, the most common procedure was appendectomy. Regarding neurosurgery, the most common was posterior spinal fusion (indicated to treat scoliosis). The detailed characteristics of the included studies, along with a description of the population and a summary of the different outcomes, are shown in Table 2.

**Table 2:** Summary and baseline data of the included studies. BMI: body mass index; NSQIP: National Surgical Quality Improvement Program; NR: not reported. Type of operation: NS: neurosurgery; ORT: orthopedics; ENT: otorhinolaryngology; GS: general surgery; UR: urology; PS: plastic surgery; CTS: cardiothoracic surgery. Outcomes: SSI: surgical site infection; WD: wound dehiscence; DVT: deep venous thromboembolism; BRT: bleeding requiring transfusion; PAR: postoperative abscess rate; LOS: length of stay; OT: operative time. Qualitative variables are reported as percentage (%), and quantitative variables as mean and standard deviation. P-values in bold indicate a statistically significant difference.

Author, year	Country	Type of study	Years evaluated	Sample size	Age: mean (SD) or range	Male %	Type of operation	Outcomes measures	Results		
									Normal weight	Obese	P-value
Delgado-Miguel et al. [6] 2020	Spain	Cohort	2017–2018	403	10.1 (3.2)	66	GS	OT	44.6 ± 18.19	57.57 ± 22.53	<0.001
								LOS	3.29 ± 2.87	3.43 ± 2.75	0.344
								SSI	4.2	10.3	0.248
								WD	2.3	7.2	0.355
								PAR	5.3	5.2	0.233
Malik et al. [7] 2019	USA	Cohort	2012–2016	1,005	10 (NR)	49	NS	SSI	4.5	7.9	0.036
								WD	18.6	25.5	0.076
								DVT	0.4	0	0.311
								BRT	77.6	73.9	0.133
Stey et al. [8] 2014	USA	Cohort	2011–2012	77,297	9.04 (NR)	56	GS	SSI	1.84	2.36	<0.001
								BRT	1.21	0.44	<0.001
								DVT	0.11	0.06	0.14
Train et al. [9] 2017	USA	Cohort	2012–2014	12,9279	9.72 (NR)	54.1	NS	OT	128.4 ± 114.1	140.4 ± 121.6	<0.001
							ORT		125.8 ± 124.4	122.1 ± 119.3	0.030
							ENT		90-6 ± 78.8	91.8 ± 83.0	0.462
							GS		70 ± 70.5	72.2 ± 66.9	0.010
							UR		109.9 ± 109.0	136.2 ± 134	<0.001
							PS		77.2 ± 79.2	81.3 ± 81.4	0.055
Kao et al. [20] 2019	USA	Cohort	2012–2015	731	14.5 (2.9)	51.8	GS	SSI	2.0	4.5	0.24
								WD	9.6	13.4	0.35
								BRT	4.5	7.8	0.33
								OT	221.6 ± 111.2	272.35 ± 122.95	<0.001
								LOS	8.5 ± 8.0	10 ± 11.9	0.04
Lavin et al. [21] 2015	USA	Cohort	2012	21,252	8.6 (NR)	58.53	ENT	BRT	10.4	5.6	<0.001
Seeley et al. [22] 2014	USA	Cohort	1999–2011	313	5.6 (NR)	58.47	ORT	SSI	0.55	0.76	NR
Blanco et al. [23] 2012	USA	Cohort	2008–2010	319	9.05 (NR)	55.2	GS	LOS	4.3 ± 3.1	4.14 ± 2.4	0.700
								SSI	5.1	4	0.800
Garey et al. [24] 2011	USA	Cohort	2005–2009	220	9.3 (NR)	59	GS	SSI	5.8	7.9	<0.001
								PAR	29	13	0.01
Davies et al. [25] 2007	Canada	Cohort	2000–2005	282	10.2 (NR)	60	GS	SSI	6.8	13.3	0.26
								PAR	3.6	3.3	0.7
Leet et al. [26] 2005	USA	Cohort	NI	103	9.3 (NR)	68.93	ORT	SSI	1.07	10	NR
								WD	0	10	NR
Alshehri et al. [27] 2018	Canada	Cohort	2012–2014	22,810	13.5 (NR)	58	GS	SSI	4.2	5.6	<0.05
								WD	0.3	0.4	NR
								BRT	2.1	1	<0.05
								DVT	0.2	0	<0.05
Chang et al. [28] 2015	Taiwan (Republic of China)	Cohort	2009–2013	97	7 (NR)	65	ORT	SSI	0	7.69	0.111
García et al. [29] 2019	USA	Cohort	2012–2017	149	14 (NR)	65	CTS	SSI	2.2	11.1	NR
Scaramuzzo et al. [30] 2023	Italy	Cohort	2011–2016	65	14.66 (2.27)	45.3	NS	OT	199.62 ± 42.23	238.9 ± 34.85	0.008
Garey et al. [31] 2010	USA	Cohort	2000–2009	247	14 (4.2)	24	GS	OT	77 ± 42	76 ± 36	0.87
								LOS	1.6 ± 1.2	1.3 ± 0.8	0.072
Shen et al. [32] 2023	China	Cohort	2017–2021	36	8 (NR)	44.4	NS	OT	200.6 ± 47.4	171.7 ± 69.2	0.22
Basques et al. [33] 2019	USA	Cohort	2012–2014	996	8 (4.6)	52.8	ORT	SSI	1.19	2.6	0.181
								DVT	0.12	0	NR
								BRT	22.77	35.71	0.008
								WD	0.24	0	NR
Pandian et al. [34] 2017	USA	Cohort	2012–2013	1,611	Range: 13–16	19.4	GS	SSI	4	10	0.054
De la Garza Ramos et al. [35] 2017	USA	Cohort	2013–2014	2,367	14.4 (1.8)	19.9	NS	SSI	0.4	1.70	0.002
								WD	0.1	1.1	0.010
								DVT	0	0.30	0.259
								OT	270 ± 90	290 ± 102	<0.001
								LOS	4.6 ± 2.5	5.1± 5.1	0.015

Results of Syntheses

The total sample comprised 259,582 patients. Among them, 39,922 (15.38%) patients were obese, and 219,660 (84.62%) were classified as normal weight. The mean age of this population was 10.7 years, and 51.5% was the average proportion of male sex.

We extracted SSI rates from 15 studies and wound dehiscence incidence rate estimates from seven studies. Bleeding requiring transfusion was reported in six studies, while only four reported on DVT and three postoperative abscess rate formation.

Surgical Site Infection

**Figure 2:**
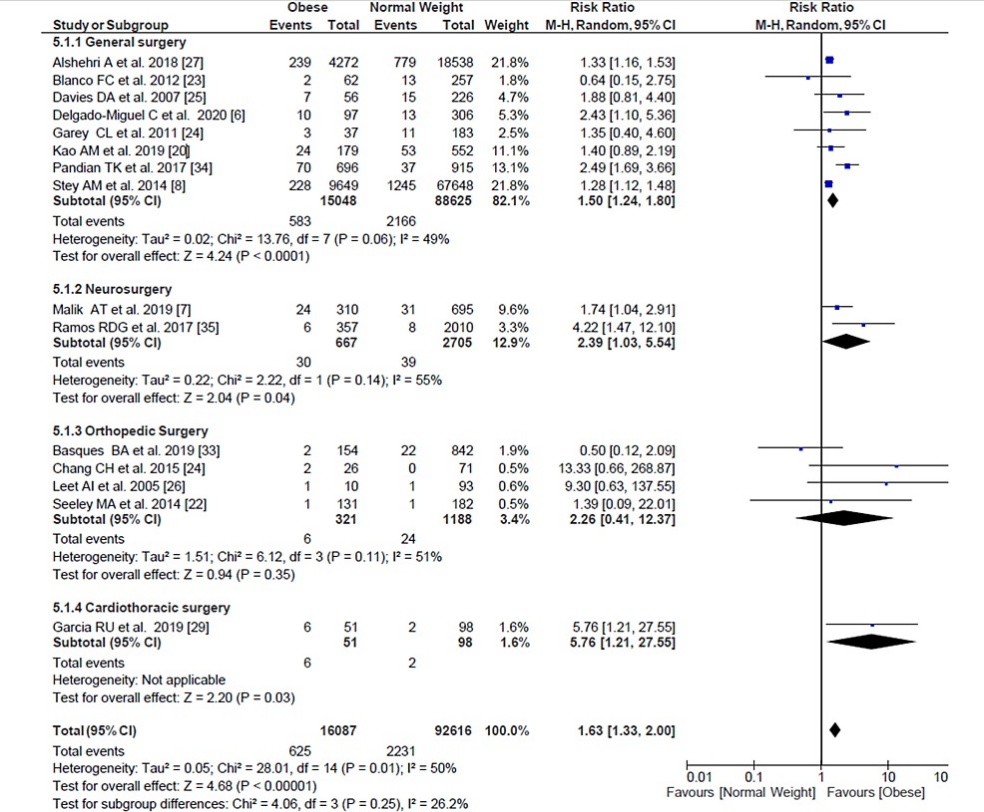
Forest plot of surgical site infection with overall risk ratio and subgroup analysis based on specialties. Delgado-Miguel et al. [6], Malik et al. [7], Stey et al. [8], Kao et al. [20], Seeley et al. [22], Blanco et al. [23], Garey et al. [24], Davies et al. [25], Leet et al. [26], Alshehri et al. [27], Chang et al. [28], García et al. [29], Basques et al. [33], Pandian et al. [34], De la Garza Ramos et al. [35].

We evaluated a pooled RR for SSI among 108,703 pediatric patients who underwent surgery, resulting in obese pediatric patients having 1.63 (95% CI = 1.33-2.00) more risk of developing an SSI than pediatric patients with normal weight (Figure 2). When analyzed by subgroups of surgical specialties, general surgery (most of them appendectomies) and neurosurgery maintained the same trend. In contrast, in orthopedic surgery, this difference was not statistically significant.

Wound Dehiscence

**Figure 3:**
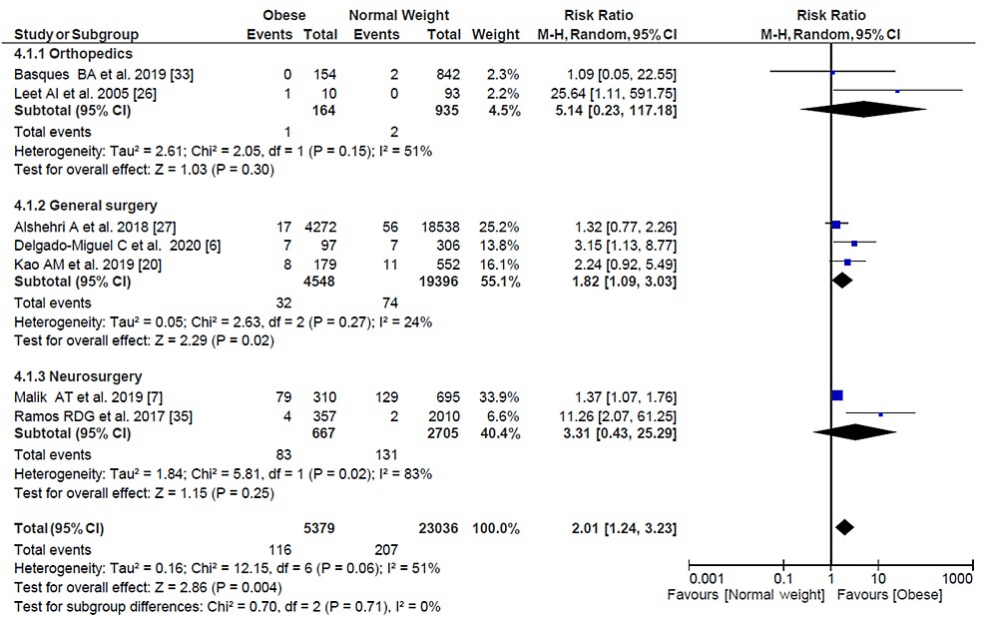
Forest plot of wound dehiscence with subgroup analysis based on specialty. Delgado-Miguel et al. [6], Malik et al. [7], Kao et al. [20], Leet et al. [26], Alshehri et al. [27], Basques et al. [33], De la Garza Ramos et al. [35].

Among the 28,415 patients from studies that considered the incidence of wound dehiscence, there was a higher risk ratio in obese patients compared with normal-weight patients (RR = 2.01, 95% CI = 1.24-3.23) (Figure 3). However, when evaluating patients in procedures outside of general surgery, the difference was not statistically significant (RR = 3.69, 95% CI = 0.80-16.98).

Bleeding Requiring Transfusion

**Figure 4:**
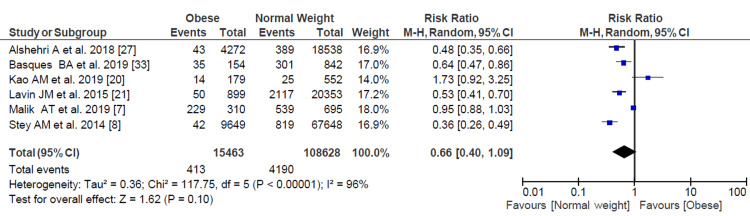
Forest plot of bleeding requiring transfusion. Malik et al. [7], Stey et al. [8], Kao et al. [20], Lavin et al. [21], Alshehri et al. [27], Basques et al. [33].

Among the 124,091 patients from studies that considered the incidence of bleeding that required transfusion during or after the surgical procedure, there was a lower risk ratio in obese patients compared with normal-weight patients. However, it was not statistically significant (RR = 0.66, 95% CI = 0.40- 1.09). The subgroup analysis between the different surgical specialties yielded similar results (Figure 4).

Deep Venous Thromboembolism

**Figure 5:**
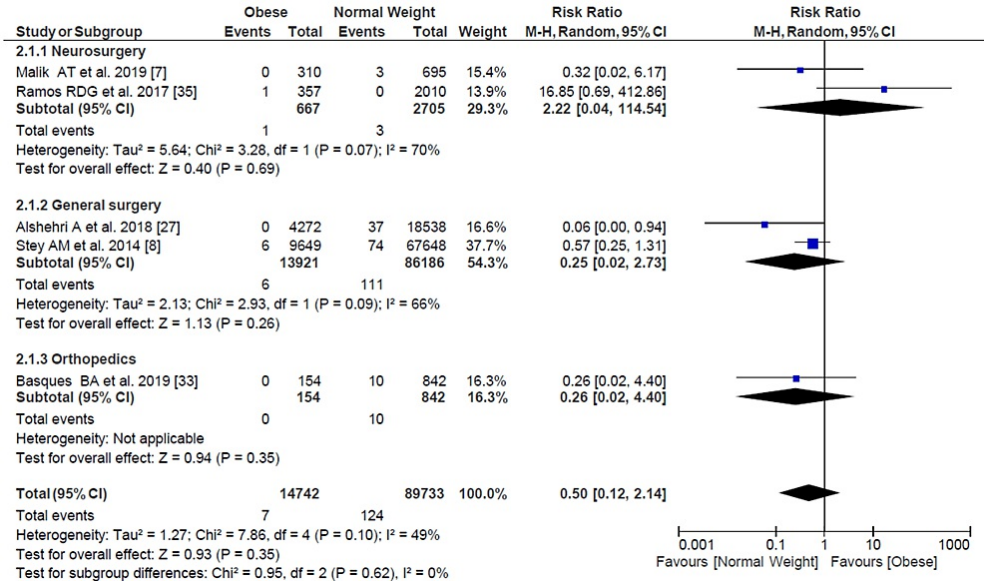
Forest plot of deep venous thromboembolism with subgroup analysis based on specialty. Malik et al. [7], Stey et al. [8]. Alshehri et al. [27], Basques et al. [33], De la Garza Ramos et al. [35].

From the four studies that reported the incidence of DVT, gathering data from 103,479 patients, there was a lower risk ratio in obese patients compared with normal-weight patients. However, it was not statistically significant (RR = 0.58, 95% CI = 0.09-3.63) (Figure 5). The subgroup analysis between the different surgical specialties yielded similar results.

Postoperative Abscess Rate

**Figure 6:**
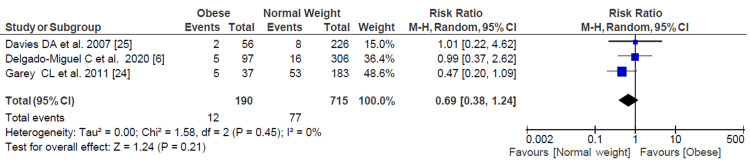
Forest plot of postoperative abscess rate. Delgado-Miguel et al. [6], Garey et al. [24], Davies et al. [25].

Only three studies reported data regarding postoperative abscess incidence with 905 patients. There was a lower risk ratio of abscess formation in obese patients compared with normal-weight patients; however, it was not statistically significant (RR = 0.69, 95% CI = 0.38-1.24) (Figure 6).

Length of Stay

**Figure 7:**
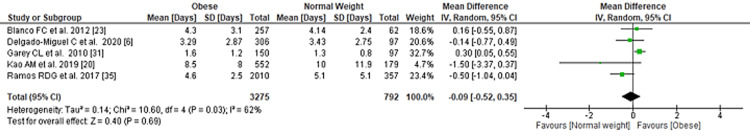
Forest plot of length of stay. Delgado-Miguel et al. [6], Kao et al. [20], Blanco et al. [23], Garey et al. [31], De la Garza Ramos et al. [35].

From five studies that gathered information from 4,067 patients, there was no difference in the LOS (days) between obese pediatric patients and normal-weight patients, as shown in Figure 7.

Operative Time

**Figure 8:**
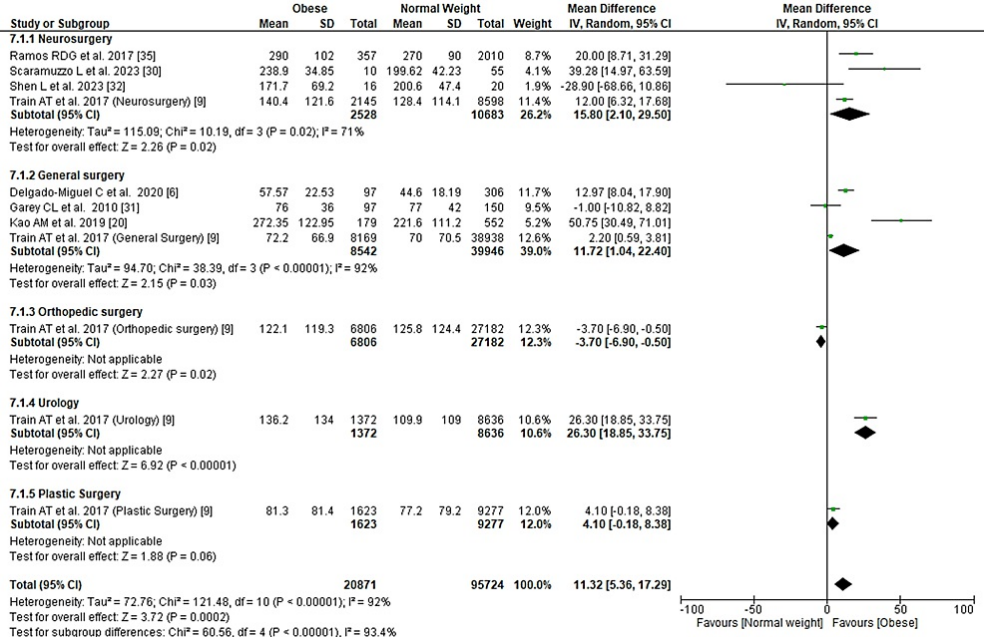
Forest plot of operative time. Delgado-Miguel et al. [6], Train et al. [9], Kao et al. [20], Scaramuzzo et al. [30], Garey et al. [31], Shen et al. [32], De la Garza Ramos et al. [35].

Seven studies presented results regarding operative time. The analyses, including information from 116,595 patients, showed that, overall, obese patients who had undergone surgery had a procedure 11.32 minutes longer than normal-weight patients, as shown in Figure 8. Train et al. [9] did not show an overall mean of operative time, instead, they presented it according to different surgical fields, including neurosurgery, orthopedic surgery, general surgery, urology, and plastic surgery. We used these subcategories along with the data from other studies.

Quality Assessment

The results of the Newcastle-Ottawa scale are shown in Table 3. Most of the studies scored 7-9, and the median score of the 20 studies was 8.1. These scores high quality according to the Agency for Health Research and Quality standards [36].

**Table 3:** Quality assessment of the included studies.

Study ID	Selection	Comparability	Outcomes	Overall (maximum 9)
Delgado-Miguel et al. [6]	4	2	3	9 (Good)
Malik et al. [7]	4	2	3	9 (Good)
Stey et al. [8]	4	2	1	7 (Fair)
TrainT et al. [9]	4	2	3	9 (Good)
Kao et al. [20]	4	2	3	9 (Good)
Lavin et al. [21]	4	2	1	7 (Fair)
Seeley et al. [22]	4	2	3	9 (Good)
Blanco et al. [23]	4	2	3	9 (Good)
Garey et al. [24]	4	2	1	7 (Fair)
Davies et al. [25]	4	2	1	7 (Fair)
Leet et al. [26]	4	2	1	7 (Fair)
Alshehri et al. [27]	4	2	3	9 (Good)
Chang et al. [28]	4	2	3	9 (Good)
García et al. [29]	4	2	1	7 (Fair)
Scaramuzzo et al. [30]	4	2	3	9 (Good)
Garey et al. [31]	4	2	1	7 (Fair)
Shen et al. [32]	4	2	1	7 (Fair)
Basques et al. [33]	4	2	3	9 (Good)
Pandian et al. [34]	4	2	1	7 (Fair)
De la Garza Ramos et al. [35]	4	2	3	9 (Good)

Heterogeneity and Risk of Bias Across Studies

**Figure 9:**
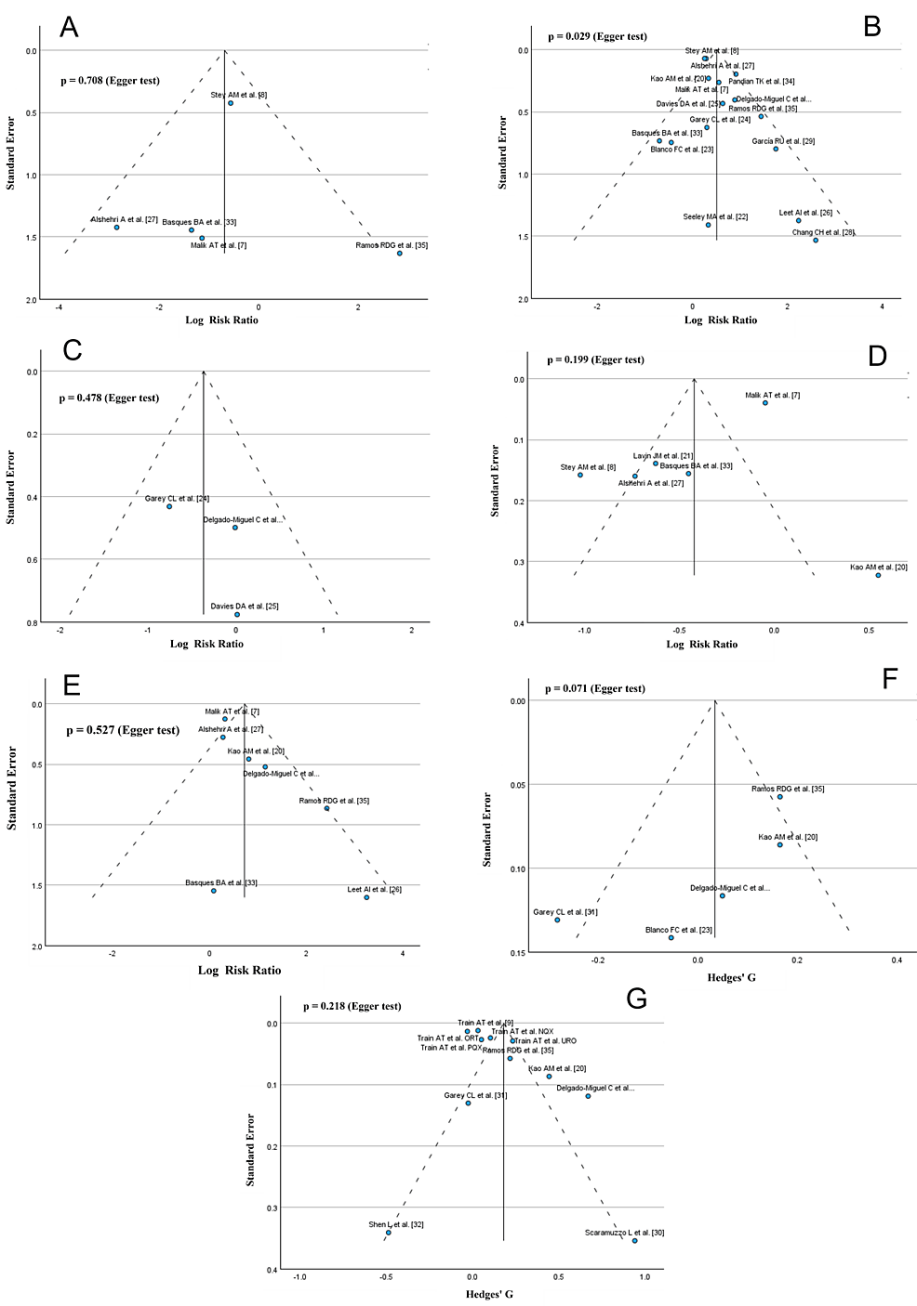
Funnel plots of the studied outcomes. The solid line represents the estimated overall effect size and the dashed line the 95% confidence interval. A: deep venous thromboembolism, B: surgical site infection, C: postoperative abscess rate, D: bleeding requiring transfusion, E: wound dehiscence, F: length of stay, G: operative time.

There was moderate to substantial heterogeneity in all the outcomes analyses (SSI: I^2^ = 50%; wound dehiscence: I^2^ = 51%; bleeding requiring transfusion: I^2^ = 96%; deep venous thromboembolism: I^2^ = 49%; postoperative abscess rate: I^2^ = 0%; LOS: I^2^ = 62%; operative time: I^2^ = 92%). The comparison-adjusted funnel plots and Egger’s test for the different outcomes are shown in Figure 9. Only for the analysis of SSI and LOS, Egger’s test was p < 0.1, suggesting the presence of publication bias in those cases.

Discussion

This meta-analysis systematically gathered relevant studies that considered the effect of obesity on intra and postoperative outcomes. After considering the data from included studies in which obese pediatric patients were compared with normal-weight patients, we can assume that the former group had a higher risk of developing SSI and wound dehiscence, in addition to having a longer operative time.

Most articles that addressed the subject of pediatric obesity in the context of surgical procedures were related to bariatric surgery [37] or, in the case of pediatric patients, in the context of the surgical management of obstructive sleep apnea [38]. Some studies presented surgical outcomes based on the pediatric patient’s weight. However, the majority of these studies focused only on specific outcomes for particular interventions, as the study presented by Patel et al. studying the impact of pediatric obesity in anterior cruciate ligament reconstruction, which demonstrated in more than 1,000 patients that children with elevated BMI had a higher rate of concurrent meniscus tears compared to those with normal BMI and 1.6 times higher odds of requiring a meniscectomy [39], the effect of obesity in the incidence of lymphocele after transplant surgery [40], or readmission after spine surgery [41], making it challenging to conclude the impact of pediatric obesity in general surgical complications. Despite how well-known the effect of obesity is in the adult population, and regardless of the high rates of obesity in the pediatric population, no meta-analysis had been conducted on the subject before.

Obesity as a risk factor for SSI has been extensively studied in the adult population. Several meta-analyses have been conducted, such as the one by Liu et al., which found that obesity increased the risk of non-superficial SSI after spinal surgery [10]. Similarly, Liu et al. reported that BMI ≥30 kg/m^2^ resulted in significantly higher SSI after colorectal surgery [42]. However, some other studies in adult populations have found no difference based on BMI for SSI, such as in the case of inguinal hernia repair [14]. In our study, encompassing different types of surgery, we found that obese pediatric patients had a 1.63 times higher risk of developing an SSI compared to normal-weight patients.

Nishimura et al. discovered changes in the expression of peripheral blood lymphocyte cytokines in obesity: transcript levels of interleukin (IL)-6, IL-1β, and tumor necrosis factor-alpha (TNF-α) were decreased, but not IL-1Ra. Serum IL-1β was undetectable, serum levels of IL-1Ra were elevated, and serum levels of TNF-α were decreased. The mechanisms that predispose obese patients to infection may involve the active participation of adipose tissue in inflammation and immunity. Obesity during surgery can lead to longer recovery times, disruption of the body’s homeostatic balance, and increased local tissue damage associated with retraction, all of which contribute to an increased risk of SSI [43,44].

Several studies have demonstrated the impact of obesity on the rates of wound dehiscence [45]. We found that obese pediatric patients had two times higher risk of developing wound dehiscence compared with normal-weight patients. The same has been reported in adults in the context of vascular procedures and laparotomies engaging the small intestines, colon, and rectum [46].

Wound dehiscence is a serious complication after surgery and is associated with increased morbidity and mortality. It has been proposed that obesity increases the risk of dehiscence, both directly, by increasing the tension in the fascial edges at the time of wound closure, and indirectly, by increasing the risk of SSI, which is also a risk factor for dehiscence [47].

In adults, obesity has been reported as a risk factor for increased operative time, such as in the study by Saiganesh et al., where they analyzed data from more than 45,000 patients who underwent open and laparoscopic ileocolic resections, partial colectomies, and low pelvic anastomoses [48]. In children, according to Garey et al., 31% of the 312 American children undergoing laparoscopic cholecystectomy at their facility were obese, yet there were no differences between the group and the normal-weight children in terms of operative time, LOS, or postoperative complications [31]. Concomitantly, Pandian et al. reported obesity was an independent predictor of operative time >90 minutes for the same procedure [34]. Finally, our study reported that, overall, obese patients who had undergone surgery had a procedure 11.32 minutes longer than normal-weight patients.

Increasing operative time is a known factor associated with increased odds of surgical complications in different types of procedures due to prolonged exposure to anesthesia, increased blood loss, more significant tissue trauma and inflammation, and a higher risk of fluid and electrolyte imbalance [49,50]. In addition to infection, longer surgeries are associated with higher rates of dehiscence, erythema, necrosis, seroma, hematoma, and delayed wound healing [51].

According to our data, other outcomes, such as LOS, are not influenced by patient weight. Similarly, in a meta-analysis conducted by Quiao et al. gathering data from more than 13,000 patients who underwent possibly curative surgery for colorectal cancer, there was no significant difference in postoperative hospital stay [52].

Higher incidence of bleeding and DVT were not found to be related to obesity in our review, in contrast with the meta-analysis by Jiang et al. in which both outcomes were significantly increased in the obese group based on the analysis of more than 90,000 patients who underwent spinal surgery [15].

For the postoperative abscess rate, which was not related to obesity according to our results, different findings have been reported in previous studies. Schlottmann et al. found that patients with obesity had a higher chance of having a postoperative intra-abdominal abscess after analyzing more than 1,300 laparoscopic appendectomies [53].

To our knowledge, this is the first meta-analysis approaching the general effect of obesity in the pediatric population for different surgical procedures, which was strengthened by gathering data on different surgical outcomes from a large population. However, the conclusions must be interpreted in the context of some limitations. There was some heterogeneity in the studies, which may cause no statistically significant results for certain outcomes. We included studies from different surgical procedures, which likely contributed to heterogeneity. Most studies did not include definitions of the surgical complications they reported. This lack of clarity affected the analysis, as it might have led to grouping different complications under the same category. Without clear definitions, it was challenging to discern potential variations, impacting the reliability of the results. Due to strict exclusion criteria and narrow inclusion criteria, some potential effects of obesity were not included; hence, a broader systematic review and meta-analysis that includes more studies should be considered. Moreover, additional databases could be used to have a greater reach.

## Conclusions

The findings of this study hold significant implications for clinical practice, suggesting the potential for additional measures to prevent surgical complications in children. However, even with supposed good-quality studies, the information is not sufficient to assume a clear recommendation. Because there are established risks, with biological explanations, in the adult population for patients with obesity undergoing surgery, it is reasonable to think that children can have similar risks. The study’s outcomes provide a foundation for reconsidering existing protocols to better address the unique challenges faced by patients with obesity. With further investigations, the consideration of segmenting antibiotic prescriptions based on BMI could be explored. Our results indicate the possibility of implementing supplementary protocols to reduce the risk of SSI or wound dehiscence in patients with obesity or reduce the operative time, which is an independent risk factor for surgical complications. As more data from different surgical procedures is being published, further meta-analyses could center on the effect of obesity for specific surgical procedures similar to those done for adults. In the hope of producing better quality evidence, clinical research should adhere to universal definitions of surgical complications. Due to the increase in childhood obesity, the funding and development of investigations focused on this population should be encouraged.
